# Mitochondrial Genome Variation after Hybridization and Differences in the First and Second Generation Hybrids of Bream Fishes

**DOI:** 10.1371/journal.pone.0158915

**Published:** 2016-07-08

**Authors:** Wei-Zhuo Zhang, Xue-Mei Xiong, Xiu-Jie Zhang, Shi-Ming Wan, Ning-Nan Guan, Chun-Hong Nie, Bo-Wen Zhao, Chung-Der Hsiao, Wei-Min Wang, Ze-Xia Gao

**Affiliations:** 1 College of Fisheries, Key Lab of Freshwater Animal Breeding, Ministry of Agriculture, Huazhong Agricultural University, Wuhan, Hubei, People’s Republic of China; 2 Freshwater Aquaculture Collaborative Innovation Center of Hubei Province, Wuhan, People’s Republic of China; 3 Department of Bioscience Technology, Chung Yuan Christian University, Chung-Li, Taiwan; SOUTHWEST UNIVERSITY, CHINA

## Abstract

Hybridization plays an important role in fish breeding. Bream fishes contribute a lot to aquaculture in China due to their economically valuable characteristics and the present study included five bream species, *Megalobrama amblycephala*, *Megalobrama skolkovii*, *Megalobrama pellegrini*, *Megalobrama terminalis* and *Parabramis pekinensis*. As maternal inheritance of mitochondrial genome (mitogenome) involves species specific regulation, we aimed to investigate in which way the inheritance of mitogenome is affected by hybridization in these fish species. With complete mitogenomes of 7 hybrid groups of bream species being firstly reported in the present study, a comparative analysis of 17 mitogenomes was conducted, including representatives of these 5 bream species, 6 first generation hybrids and 6 second generation hybrids. The results showed that these 17 mitogenomes shared the same gene arrangement, and had similar gene size and base composition. According to the phylogenetic analyses, all mitogenomes of the hybrids were consistent with a maternal inheritance. However, a certain number of variable sites were detected in all F_1_ hybrid groups compared to their female parents, especially in the group of *M*. *terminalis* (♀) *× M*. *amblycephala* (♂) (*MT*×*MA*), with a total of 86 variable sites between *MT*×*MA* and its female parent. Among the mitogenomes genes, the protein-coding gene *nd5* displayed the highest variability. The number of variation sites was found to be related to phylogenetic relationship of the parents: the closer they are, the lower amount of variation sites their hybrids have. The second generation hybrids showed less mitogenome variation than that of first generation hybrids. The non-synonymous and synonymous substitution rates (dN/dS) were calculated between all the hybrids with their own female parents and the results indicated that most PCGs were under negative selection.

## Introduction

Mitochondria not only provide energy for animal cells [1], but also play a crucial role in the dynamics of molecular evolution, species hybridization, gene introgression and species differentiation [2]. Mitochondrial genome (mitogenome) has been extensively used to evaluate genetic diversity of different populations in fish species and analyze genetic characteristics of the hybrid fishes due to its small molecular weight, maternal inheritance, relatively rapid substitution rate, and lack of recombination [2]. Guo et al. [3] proved that the *atpase8* and *atpase6* genes are useful genetic markers to monitor the variations in the hybrid progeny among the different carp strains. Avise and Saunders [4] took advantage of polymorphisms in mitochondrial DNA (mtDNA) to analyze hybridization and introgression among sunfish species (*Lepomis*, Centrarchidae).

Although mitochondrial DNA is usually maternally inherited, some studies also reported the variation of mitogenome sequences between hybrids and their female parents. In mammals, the studies in murine hybrids and the interspecies cross between the domestic cow and the Asian wild gaur proved the evidences of paternal inheritance and recombination of mtDNA [5–7]. These results indicated that maternal inheritance of mitochondrial genome may involve species specific regulation and can be disrupted by hybridization. Although many studies demonstrated that mtDNA of hybrid fishes followed strict rules of maternal inheritance [3, 8–13], variations of mitochondrial DNA between hybrids and their female parents have also been reported in some fish species. For example, Guo et al. [14] found that complete mtDNA nucleotide identity between the triploid crucian carp and its male parent allotetraploid was higher than that between the triploid crucian carp and its female parent Japanese crucian carp (98% and 93%, respectively).

There are six bream fish species distributed in Chinese natural lakes or rivers, including *Megalobrama amblycephala*, *M*. *skolkovii*, *M*. *terminalis*, *M*. *pellegrini* and *M*. *elongata* belonging to *Megalobrama* genus, as well as white Amur bream (*Parabramis pekinensis*) in *Parabramis* genus, the sister genus of *Megalobrama* [15, 16]. Because of their economically valuable traits, bream fishes have been considered as main aquaculture fish species in China since 1960s [17]. In order to breed superior culture strains, hybridization has been conducted among these fish species [18]. Xie et al. [19] conducted the hybrid breeding of *M*. *hoffmanni* (♀) × *M*. *amblycephala* (♂) and discovered that the first generation hybrid featured high survival rate and was fertile. It resembled *M*. *hoffmanni* in flesh quality and had advantages over *M*. *hoffmanni* in resistance to hypoxia. The previous study had also detected that the hybrids of *M*. *amblycephala* (♀) × *P*. *pekinensis* (♂) exhibited better disease resistance [20].

In this study, we sequenced the complete mitogenomes of 7 hybrid groups of the bream species for the first time. Along with the 10 mitogenomes reported previously by our group, a total of 17 mitogenomes were analyzed in the study, representing 5 bream species, 6 first generation hybrids and 6 second generation hybrids. We explored the way the mitogenomes are inherited during hybridizations among different bream species and how the variability of the studied mitogenomes is distributed in mitochondrial genes of hybrids in comparison to their parental species.

## Materials and Methods

### Ethics statement

All experiments were conducted following the ‘‘Guidelines for Experimental Animals” of the Ministry of Science and Technology (Beijing, China). The study was approved by the Institutional Animal Care and Use Ethics Committee of Huazhong Agricultural University. All efforts were made to minimize animal suffering.

### Samples and DNA extraction

The parents of each crossing combination were all from wild population and the hybrid breeding artificial reproduction was conducted in 2012 (first generation, F_1_) and 2015 (second generation, F_2_) in Fish Breeding Base of College of Fisheries (Hubei Bai Rong Improved Aquatic Seed CO., LTD, Huanggang, 438800), Huazhong Agricultural University, Hubei province of China. Specimens of hybrid offspring were collected randomly from their population. Total genomic DNA was extracted from the fin tissue using a modified ammonium acetate precipitation protocol [21]. The bream fish types and cross combinations are showed in Table 1. Ten individuals from each group were used for the study.

**Table 1:** The fish types, their simplified name used in the paper and the accession number of their mitogenomes as submitted to Genebank.

Species	Simplified name	Accession number
*Megalobrama amblycephala*	*MA*	NC_010341.1
*Megalobrama terminalis* or *Megalobrama hoffmanni*	*MT*	JX242530
*Megalobrama skolkovii*	*MS*	JX242528
*Megalobrama pellegrini*	*MP*	JX242529
*Parabramis pekinensis*	*PP*	JX242531.1
*Megalobrama amblycephala* (♀)*×Parabramis pekinensis* (♂)	*MA*×*PP*	KF927167.1
*Megalobrama amblycephala* (♀)*×Megalobrama terminalis*(♂)	*MA*×*MT*	KP025957
*Megalobrama amblycephala* (♀)*×Megalobrama skolkovii* (♂)	*MA*×*MS*	KT347220
*Megalobrama amblycephala* (♀)*×Megalobrama pellegrini* (♂)	*MA*×*MP*	KT851551 (this study)
*Megalobrama terminalis* (♀) *×Megalobrama amblycephala* (♂)	*MT*×*MA*	KP772253
*Megalobrama skolkovii* (♀)*×Megalobrama amblycephala* (♂)	*MS*×*MA*	KT316879
(*MA*×*MT*) (♀)×*MA* (♂)	(*MA*×*MT*)×*MA*	KT851547 (this study)
(*MA*×*PP*) (♀)×*MA* (♂)	(*MA*×*PP*)×*MA*	KT851549 (this study)
(*MA*×*PP*) (♀)×(*MA*×*PP*) (♂)	(*MA*×*PP*)×(*MA*×*PP*)	KT851552 (this study)
(*MA*×*MT*) (♀)×(*MA*×*MT*) (♂)	(*MA*×*MT*)×(*MA*×*MT*)	KT851553 (this study)
(*MA×MT*) (♀)×(*MA*×*PP*) (♂)	(*MA*×*MT*)×(*MA*×*PP*)	KT851545 (this study)
(*MA*×*PP*) (♀)×(*MA*×*MT*) (♂)	(*MA*×*PP*)×(*MA*×*MT*)	KT851550 (this study)

### Primer design, PCR amplification and sequencing

Twenty-four pairs of PCR primers (Table 2) were designed to amplify the whole mitogenome sequences based on the conserved sequences from *M*. *amblycephala* (GenBank NC_010341.1), using Primer 6.0 and Oligo 7.0 software. The primers were synthesized by Life Technologies Biotechnology Company (Shanghai, China). The amplifications were performed in 25 μL reaction volume containing 1× LA PCR buffer II (Mg^2+^), 1.25 mM of dNTPs, 0.5 mM of each primer, 1.25 U of LA *Taq* polymerase (Takara, Dalian, China), approximately 100 ng of template genomic DNA. PCR was performed under the following conditions: denaturation at 95°C for 5 min, followed by 30 cycles at 98°C for 10 s, 52–58°C for 45 s and 72°C for 1 min, as well as further incubation for 10 min at 72°C. Subsequently, the PCR products were purified and directly sequenced by Quintarabio Biotechnology Company (Wuhan, China).

**Table 2:** Primers designed for amplifying mitogenomes.

Primer name	Primer sequence(5’-3’)
primer1H721	CAGCGAATCCTATTATCCTTGTC
primer1L2008	GGTGTAAGTGAGATGCTTGAC
primer2H1744	CTATCACAGAACACTACGAACA
primer2L3104	AGCCATTCATACAGGTCTCTAT
primer3F	TCTTCTCCAAGCACAAGTGTA
primer3R	GGAATAGTACGGCTGATAAGGT
Hmt4F	GACCACTAGCCGCAATATGATAT
Hmt4R	GGTTGTTGTTAGGACTGGACTT
primer5H5549	ATCGCACACATAGGCTGAAT
primer5L7022	GGAGAAGAAGTACGGCTGTTA
primer6F	GAGCATCCGTAGACCTAACAAT
primer6R	GTGAGGCAATGAAGGCAGTT
primer7F	AACAGACCACCGAATAGTAGT
primer7R	GAAGACAGACAGCCAGGAAT
primer8H10194	CCACCACAGCATCATAGAAGG
primer8L11884	TTAGGAGTATCGGTTGAGAGTATTG
primer9H11580	CGAACCTATCAGCCGACAAC
primer9L12879	TTCCTAAGACCAATGGATGAACTG
primer10H12521	CGTTAATTACAGCAGGCTACTC
primer10L13587	GCGAGGATTAGTCCGATTAGG
primer11H13357	CTGAGAAGGTGTCGGAATTATATC
primer11L14881	TGTTAGTGGTGTGGCTGTTAT
Hm12F	CCATAGAACTTACAGCCATAACC
Hm12R	TTACGGATGAGTCAACCATAGT
primer13H15302	CTTGAAGAACCACCGTTGTAGT
primer13L16436	GAGGATGAGGAATAATGCGAAGTA
primer14H16170	TTGCTTACGCCATTCTACGA
primer14L978	CATCTTCAGTGCTATGCTTTGT
DloopF	TGGCTTCAATCTCAGGAACAT
DloopR	ACATCTTCAGTGCTATGCTTTG
gap1F	TCACAGAACACTACGAACAT
gap1R	TGGACCTCCTATACTCAGTT
gap2F	CTGTGGCAATTACACGCTGATT
gap2R	AGGACTGGTAGAGATAAGAGAAGAAG
gap3F	TGAACCTATCAGCCGACAAC
gap3R	CCTAAGACCAATGGATGAACTG
gap4F	AACCGAGACCAGTGACTTGAAGAAC
gap4R	GGATGGATCGTAGAATGGCGTAAGC
gap5F	ACCCGCATTCGTTCAAGT
gap5R	TTACGGTGGCAAGTCATAGTG
gap6F	CACTCGGCTACCCTACCTGT
gap6R	TTCCTGTCAATCCACCCAC
hybridCOX1F	GCTGATGATAAGGACAGGA
hybridCOX1R	GTAGTATGTGTCGTGAAGG
hybridATP6F	CGTTCCATCTCTAGGTGTA
hybridATP6R	GTGAGGAGTAGAAGGATTATC
hybridND6F	TAGAACTTACAGCCATAACC
hybridND6R	TTACGGATGAGTCAACCA

### Gene annotation and sequence analysis

After sequencing, the sequence fragments were edited by DNASTAR 5.0 software to obtain the complete mitogenome sequences. Annotation of protein-coding genes (PCGs), ribosomal RNA genes, transfer RNA genes and definition of their respective gene boundaries were performed by MitoAnnotator software (http://mitofish.aori.u-tokyo.ac.jp/annotation/input.html). The secondary structures of the tRNA were predicted by tRNAscan-SE1.21 software [22]. Sequence alignment was carried out with the Clustal W (http://www.ebi.ac.uk/Tools/msa/clustalw2/). In order to know the selective pressure acted on each protein-coding gene in these species, we calculated the non-synonymous and synonymous substitution rates (dN/dS) using YN00 in PAML software [23, 24] between all the hybrids with their own female parents.

### Phylogenetic analyses

Together with these 17 breams, three out-group species, *Ancherythroculter nigrocauda* (KC513573.1), *Culter alburnus* (KM044500.1) and *Chanodichthys mongolicus* (KC701385.1) were included in our analysis. Phylogenetic tree building was conducted with Neighbor-Joining (NJ) analyses using MEGA 5.0 software [25]. Due to the importance of protein-coding genes (PCGs) in inferring species phylogeny, our phylogenetic analyses were based on the nucleotide sequences of 13 PCGs from 20 mitogenomes.

## Results and Discussion

### Phylogenetic relationship

**Fig 1:**
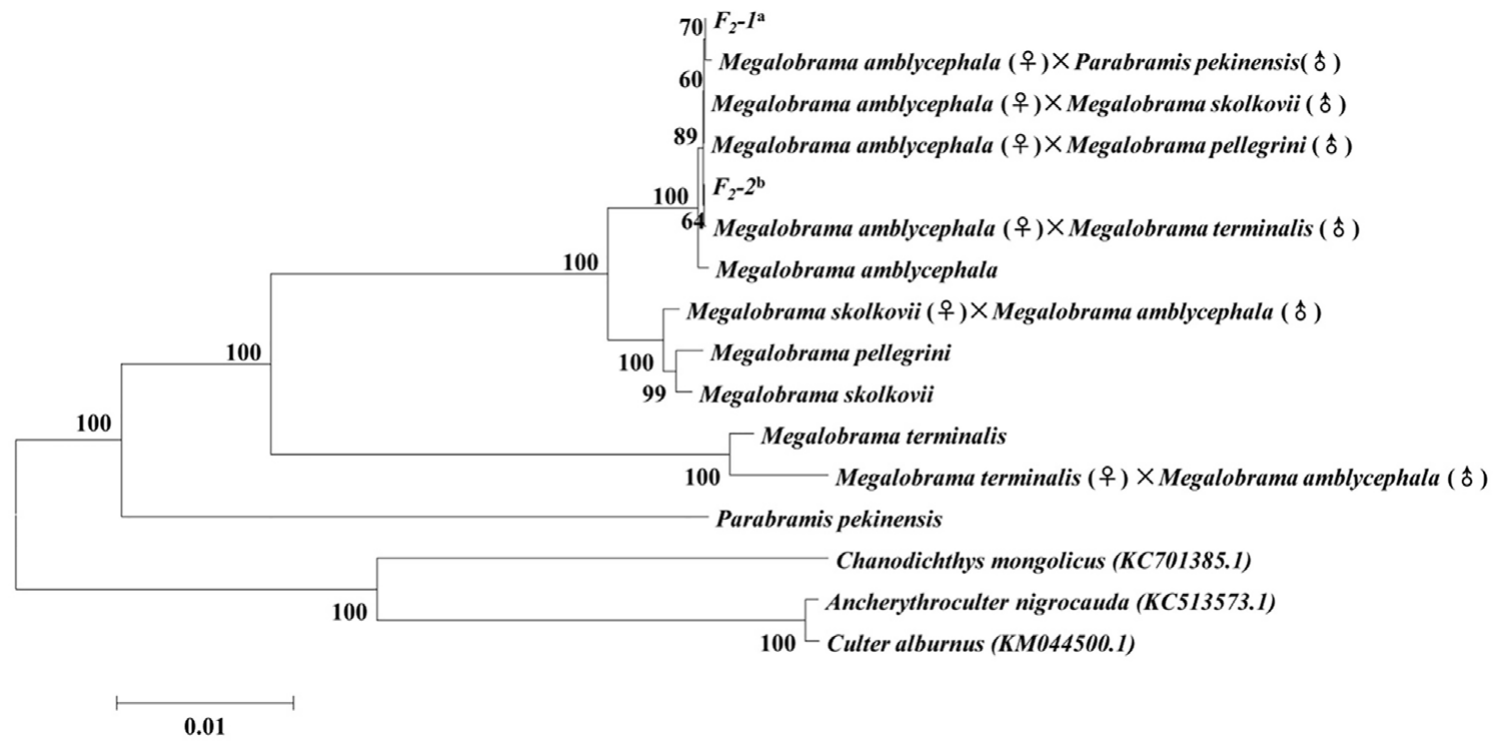
The phylogenetic trees based on the nucleotide sequences of 13 protein-coding genes from 20 mitogenomes using the NJ method. ^a^*F*_*2*_*-1* represents F_2_ hybrids of (*MA***×***MT*)**× (***MA***×***PP*), (*MA***×***PP*)**×***MA*, (*MA***×***PP*)**×(***MA***×***MT*), (*MA***×***PP*)**×**(*MA***×***PP*) and (*MA***×***MT*)**×**(*MA***×***MT*); ^b^*F*_*2*_*-2* represents F_2_ hybrids of (*MA***×***MT*)**×***MA*. Numbers above the nodes represent bootstrap values.

According to the phylogenetic tree (Fig 1), all bream fishes and their hybrids were clustered together. The three outgroup species (*A*. *nigrocauda*, *C*. *alburnus* and *C*. *mongolicus*) were grouped into a single clade. The phylogenetic analyses clustered the 4 bream fishes from the genus *Megalobrama* and 1 from the genus *Parabramis* into the same clade, which confirmed the close taxonomic relationship between these two genera. Among the *Megalobrama* species, *MS* and *MP* have the closest phylogenetic relationship, with *MA* being more closely related to them than *MT*. This result is consistent with the traditional taxonomic relationship of these species reported in previous studies [26, 27]. According to the mitogenomes, each hybrid (F_1_ and F_2_ groups) was more closely related to its own female parent.

### Mitogenome organization and composition

**Fig 2:**
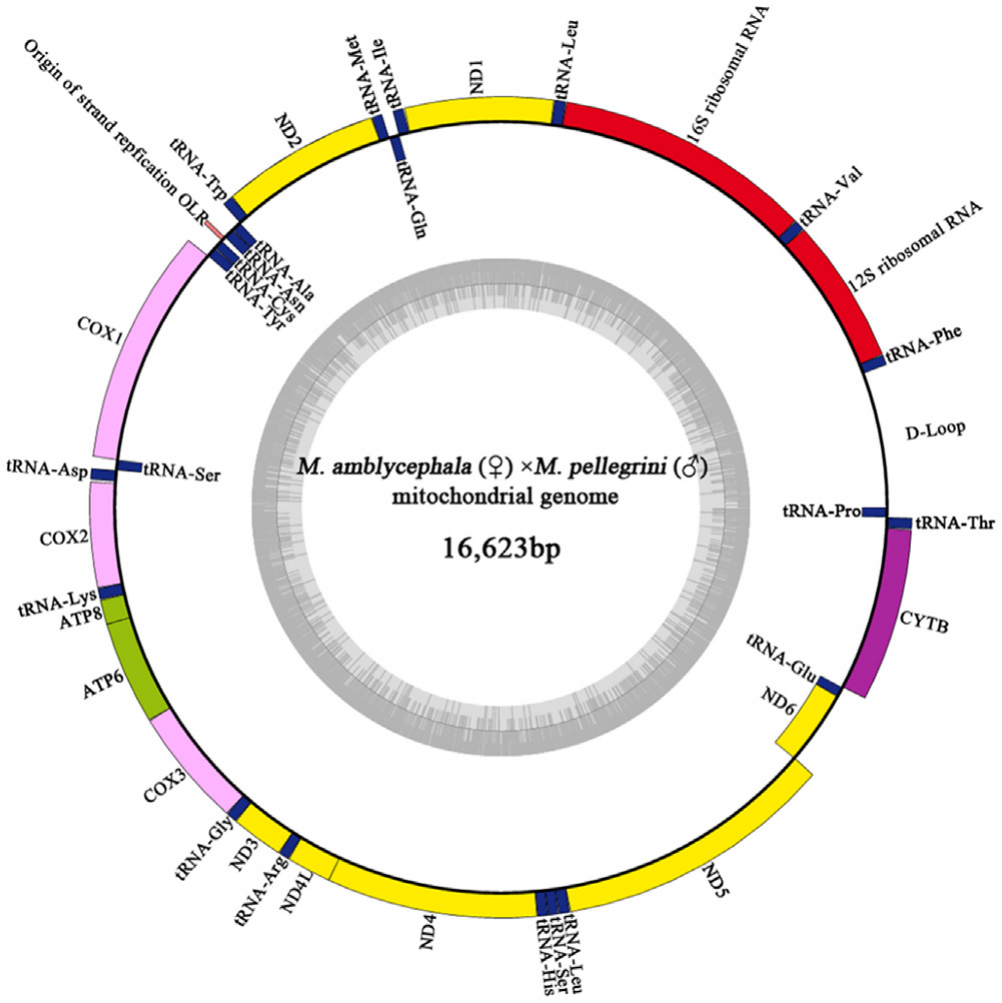
Gene map of *Megalobrama amblycephala* (♀) × *Megalobrama pellegrini* (♂) mitogenome. All the bream species and the hybrid individuals shared the same gene arrangement and possessed similar gene sizes. Those genes encoded on H/L-strand are shown outside/inside the circular gene map, respectively. The Inner ring indicates the GC content. The figure was initially generated with OGDRAW (http://ogdraw.mpimp-golm.mpg.de/) and modified manually.

Genome length, AT-richness and base composition of the 17 mitogenomes are reported in Table 3. The lengths of the complete genome were all in the range of 16,621 to 16,623 bp, and the genome lengths of the hybrids corresponded with that of the female parents. The overall base composition was slightly AT-rich (Table 3). Each genome contained the same 22 transfer RNA genes, 13 PCGs, 2 ribosomal RNA genes, and 2 main non-coding regions of the control region and the origin of the light strand replication. Most genes of all 17 mitogenomes were encoded on heavy strand (H-strand) except for 1 PCG (the *nd6* gene) and 8 tRNA genes (tRNA-Gln, tRNA-Ala, tRNA-Asn, tRNA-Cys, tRNA-Tyr, tRNA-Ser, tRNA-Glu, and tRNA-Pro), which were encoded on light strand (L-strand) (Fig 2). Structurally, all mitogenomes tested in this study shared the same gene arrangement and also displayed conserved genomic arrangement with other teleost species [28–30].

**Table 3:** Base composition of 17 mitogenome sequences.

Species	Total length (bp)	A%	T%	G%	C%	(A+T)%
*MA*	16623	31.22	24.68	16.20	27.90	55.90
*MT*	16622	31.18	24.88	16.18	27.76	56.06
*MS*	16621	31.21	24.72	16.19	27.88	55.93
*MP*	16621	31.26	24.69	16.16	27.89	55.95
*PP*	16622	31.07	24.76	16.32	27.85	55.83
*MA***×***PP*	16623	31.24	24.69	16.19	27.88	55.93
*MA***×***MT*	16623	31.24	24.69	16.18	27.88	55.93
*MA***×***MS*	16623	31.23	24.69	16.19	27.89	55.92
*MA***×***MP*	16623	31.23	24.68	16.19	27.89	55.91
*MT***×***MA*	16622	31.13	24.94	16.21	27.72	56.07
*MS***×***MA*	16621	31.21	24.74	16.18	27.87	55.95
(*MA***×***MT*)**×***MA*	16623	31.24	24.68	16.18	27.90	55.92
(*MA***×***PP*)**×***MA*	16623	31.23	24.69	16.19	27.89	55.92
(*MA***×***PP*)**×** (*MA***×***PP*)	16623	31.23	24.69	16.19	27.89	55.92
(*MA***×***MT*)**×** (*MA***×***MT*)	16623	31.23	24.69	16.19	27.89	55.92
(*MA***×***MT*)**×** (*MA***×***PP*)	16623	31.23	24.69	16.19	27.89	55.92
(*MA***×***PP*)**×** (*MA***×***MT*)	16623	31.23	24.69	16.19	27.89	55.92

### Sequence similarity and variation

Sequence comparisons of the mitogenomes between the hybrids and their female parents revealed sequence similarities above 99% in all cases (Table 4). In the F_1_, the highest similarity (99.8797%) was detected between the *MA***×***MP* hybrid and its female parent *MA*, while the lowest similarity (99.4826%) was found between *MT***×***MA* hybrid and its female parent *MT*. A certain number of variable sites were detected between all F_1_ hybrids and their respective female parent. The results showed that reciprocal crosses between two breams can generate hybrids with different degrees of mitogenome variation. *MT***×***MA* had 86 variable sites, while the reciprocal cross *MA***×***MT* only had 23 variable sites. *MA***×***MS* had fewer variable sites (21) than *MS***×***MA* (38). There is now a broad consensus that reciprocal crosses between species can yield hybrids with different performance [31, 32]. In centrarchid fishes, of the 18 species pairs with reciprocal crosses, 17 pairs showed asymmetrical viabilities [31]. The hybrids of *Pogonias cromi* (♀) × *Sciaenops ocellatus* (♂) were found to be viable, while the fertilization rate for the reciprocal hybrids from *S*. *ocellatus* (♀) × *P*. *cromi* (♂) was 0% [33]. Kim et al. [34] had reported that the hatching rates were significantly different in the reciprocal crosses between Japanese flounder (*Paralichthys olivaceus*) and spotted halibut (*Verasper variegatus*). Nuclear–cytoplasmic interaction (especially the interaction between mitochondria and nucleus) was regarded as an important cause for asymmetric performance during reciprocal crosses [32, 35]. Whether the different variations of mitogenomes detected in the present study could be related to some effects of reciprocal hybrids among bream species, for example to how mito-nuclear interaction works different in the reciprocal hybrids, needs further investigation.

**Table 4:** Comparative analyses of sequence similarity and variable sites.

	Species	Sequence similarity	Variable sites
F_1_ groups	*MA*×*PP*	99.8496%	25
	*MA*×*MT*	99.8616%	23
	*MA*×*MS*	99.8737%	21
	*MA*×*MP*	99.8797%	20
	*MT*×*MA*	99.4826%	86
	*MS*×*MA*	99.7714%	38
F_2_ groups	(*MA*×*MT*)×*MA*	99.9880%	2
	(*MA*×*PP*)×*MA*	99.9759%	4
	(*MA*×*PP*)×(*MA*×*PP*)	99.9759%	4
	(*MA*×*MT*)×(*MA*×*MT*)	99.9759%	4
	(*MA*×*MT*)×(*MA*×*PP*)	99.9759%	4
	(*MA*×*PP*)×(*MA*×*MT*)	99.9759%	4

The variation of mitogenomes in F_1_ hybrids with *M*. *amblycephala* as female parent was distinctly lower than those with *M*. *terminalis* or *M*. *skolkovii* as female parent, which might indicate that *M*. *amblycephala* mitogenome interacts better with a hybrid nuclear background when compared to *M*. *terminalis* and *M*. *skolkovii*. In addition, there were more mutation sites in *MT***×***MA* than *MS***×***MA*. This is in accordance with their genetic relationship, as *MA* has closer phylogenetic relationship with *MS* than with *MT* [26, 27]. These results may indicate that the genetic distances between parents may have an impact on the mitogenomes of the hybrids, maybe allowing a more stable mitogenome inheritance when the hybrid nucleus derives from closer related species.

Regarding F_2_ generation hybrids, alignment analysis revealed great similarity among the 6 groups’ mitogenomes (Table 4). The mitogenome sequences of (*MA***×***PP*)**×***MA*, (*MA***×***PP*) **×**(*MA***×***PP*), (*MA***×***MT*)**×**(*MA***×***MT*), (*MA***×***MT*)**×**(*MA***×***PP*) and (*MA***×***PP*)**×**(*MA***×***MT*) were completely the same. The highest sequence similarity occurred between th*e* (*MA***×***MT*)**×***MA* and its female parent, with 2 variable sites (1 in 16s RNA and 1 non-synonymous mutation in the *nd6* gene). Four variable sites were observed in (*MA***×***PP*)**×**(*MA***×***PP*) and (*MA***×***MT*)**×**(*MA***×***MT*) compared to their female parents, which were generated by the self-cross of *MA***×***PP* and *MA***×***MT*, respectively. There was no obvious difference between the hybrids obtained by reciprocal crosses (Table 4). Compared with the variation in F_1_ groups, F_2_ groups had relatively fewer variable sites. All the results mentioned above reflect that hybridization has a weaker influence on the mitogenome sequences in F_2_ than in F_1_, maybe related to the fact that both the parents of the F_2_ derived from *MA* strains.

### Protein-coding genes

**Fig 3:**
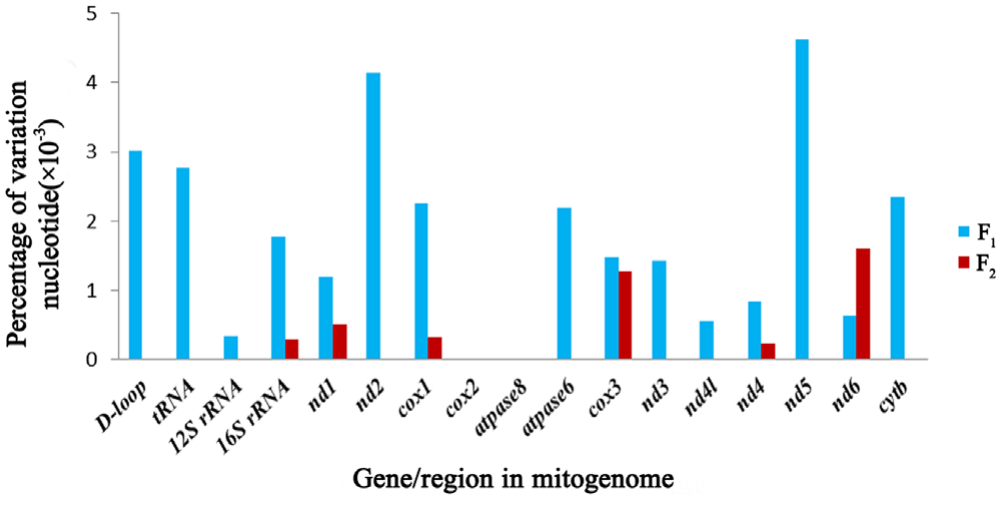
Percentage of nucleotide variation in different genes/regions of mitogenomes in F_1_ and F_2_. There were no variation site found in the *cox2* and *atpase8* genes of F_1_ hybrids, and the Dloop region, tRNA, 12S rRNA, *nd2*, *cox2*, *atpase8*, *atpase6*, *nd3*, *nd4l*, *nd5* and *cytb* genes of F_2_ hybrids. The variation rates here refer to the average variation rates.

Of the 213 variable sites found in the F_1_ groups, 150 (70.42%) were located in the mitochondrial PCGs. The *nd5* gene displayed the highest variability, with 6 non-synonymous mutations and 45 synonymous mutations. On the other hand, the *cox2* and *atp8* showed the lowest variation rates, with no variation sites found. The highest variability occurred in the *nd6* gene in the F_2_ groups (Fig 3), while there were no variable sites found in the *nd2*, *cox2*, *atpase8*, *atpase6*, *nd3*, *nd4l*, *nd5* and *cytb* genes.

**Fig 4:**
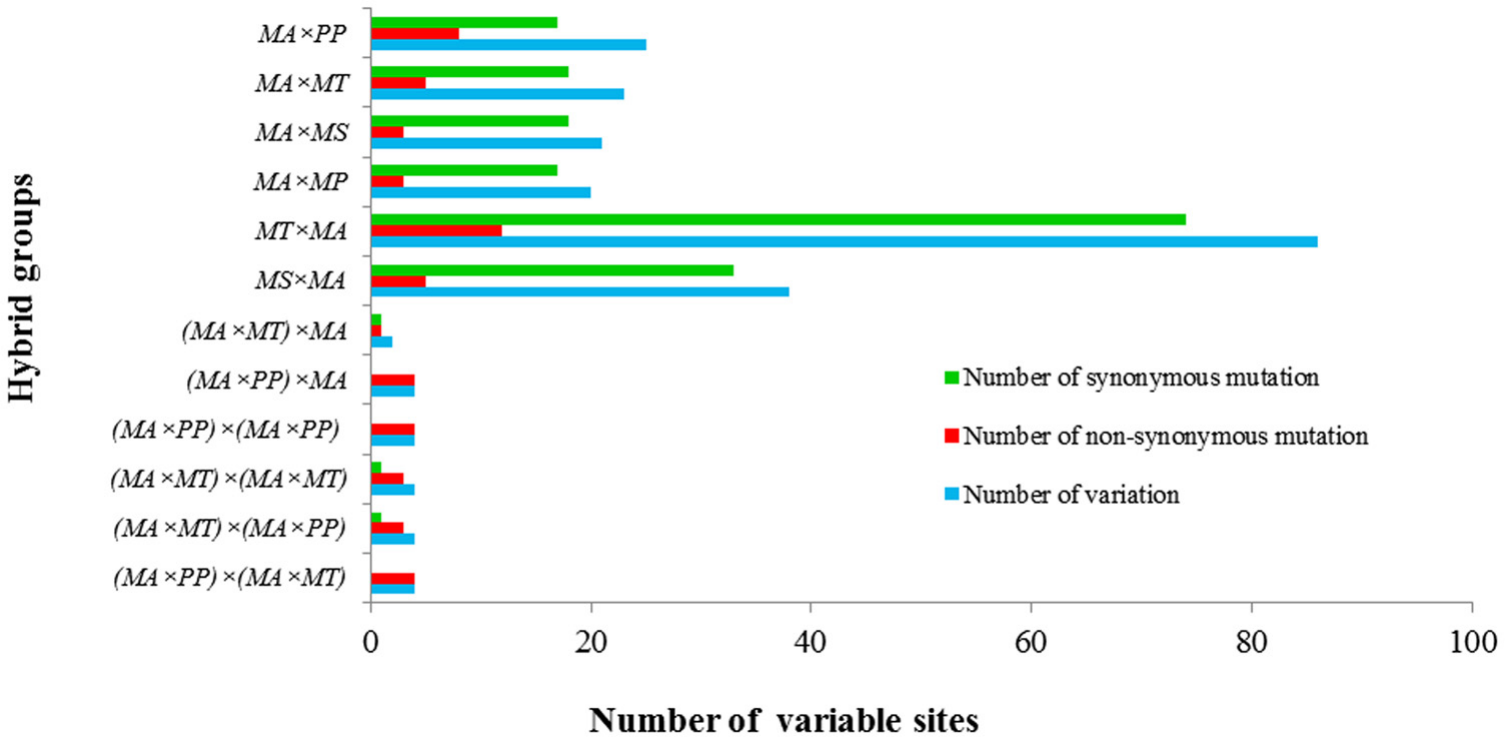
Synonymous and non-synonymous mutations of different hybrids in F_1_ and F_2_. There were no synonymous mutations found in (*MA***×***PP*)**×***MA*, (*MA***×***PP*)**×**(*MA***×***PP*) and (*MA***×***PP*)**×**(*MA***×***MT*).

Like other vertebrate mtDNAs [36, 37], most variable sites were at the third codon position, resulting in synonymous mutations (Fig 4). The non-synonymous/synonymous rate ratio (dN/dS) is widely used as an indicator of selective pressure at the sequence level among different species. It is commonly accepted that dN > dS, dN = dS, and dN < dS generally indicate positive selection, neutral mutation, and negative selection, respectively [23]. In the present study, all dN/dS values of the most PCGs (except the *cox2* and *atp8* genes) are less than 1 between F_1_ hybrids with its own female parent (Table 5), which indicates that these genes are under negative selection. Among the 13 PCGs, the highest dN/dS value was detected for the a*tp6* gene (0.4738) in the four groups (*MA*×*PP—MA*, *MA*×*MT—MA*, *MA×MS—MA* and *MA×MP—MA*) (Table 5). All the 19 variable sites found in 13 PCGs of all the F_2_ hybrids were non-synonymous mutations; therefore, the dN/dS was not calculated between the F_2_ hybrids and their own female parents. Non-synonymous substitutions are more strongly affected by natural selection than synonymous substitutions and fixations of slightly deleterious mutations are expected to increase the non-synonymous substitution rate [38]. There may be a fixation of slightly deleterious mutations, leading to an increase of non-synonymous substitution in F_2_ hybrids.

**Table 5:** The dN/dS values between the F_1_ hybrids and their own female parents.

Gene	*MA*×*PP-MA*	*MA*×*MT-MA*	*MA×MS-MA*	*MA×MP-MA*	*MT×MA-MT*	*MS×MA-MS*
*nd1*	NA^b^	NA^a^	NA^a^	NA^a^	0.4230	0.0000^c^
*nd2*	0.0000^c^	0.0000^c^	0.0000^c^	0.0000^c^	0.2207	0.0000^c^
*cox1*	0.3950	0.1973	0.1973	0.1973	0.0000^c^	0.3944
*cox2*	NA^a^	NA^a^	NA^a^	NA^a^	NA^a^	NA^a^
*atp8*	NA^a^	NA^a^	NA^a^	NA^a^	NA^a^	NA^a^
*atp6*	0.4738	0.4738	0.4738	0.4738	0.0000^c^	NA^a^
*cox3*	NA^b^	NA^a^	NA^a^	NA^a^	0.0000^c^	0.0000^c^
*nd3*	NA^a^	NA^a^	NA^a^	NA^a^	0.0000^c^	0.0000^c^
*nd4l*	NA^a^	NA^a^	NA^a^	NA^a^	NA^a^	NA^b^
*nd4*	NA^b^	NA^a^	NA^a^	NA^a^	0.1363	0.0000^c^
*nd5*	NA^a^	NA^a^	NA^a^	NA^a^	0.0244	0.1937
*nd6*	NA^a^	NA^b^	NA^a^	NA^a^	NA^a^	NA^a^
*cytb*	NA^a^	NA^a^	NA^a^	NA^a^	0.0686	0.0000^c^

^a^NA means these genes showed no variable sites between the hybrids and their own female parents;

^b^NA means there were no synonymous substitutions (dS = 0);

^c^ 0.0000 means there were no nonsynonymous substitution (dN = 0).

Some previous studies supported that mitochondrial variants may contribute to phenotypic variation in poultry and livestock. In cattle, mtDNA variation has been associated to economic traits such as milk yield, calving rates, weight and so on [39–41]. Fernandez et al. [42] justified the use of the polymorphism in the *cytb* gene as a marker for maintaining an adequate intramuscular fat level in Iberian pigs. In the present study, the *nd5* gene displayed the highest variability in the F_1_ groups. Notably, 48 variation sites were found in the *nd5* gene between *MT*×*MA* and its female parent *MT* in the present study. The polymorphism of the mtDNA *nd5* gene had been reported to be significantly associated with growth traits at 6 months in the cattle [43]. Sutarno et al. [40] also found a significant association between calving rate and mitochondrial polymorphisms of two regions (the D-loop and the *nd5* gene) in two cattle breeds. What is the likely cause of the association of mtDNA *nd5* gene polymorphism with phenotypes? Firstly, the mutation rate of mtDNA is higher than that of nuclear DNA [2]. The rapid rate of mutation in mtDNA makes it possible to produce advantageous or disadvantages phenotypes in a relatively short time [43]. Secondly, genetic variation affecting trans-acting nuclear factors can alter the mtDNA sequence and affect phenotypes, so associations between some nuclear genomic regions and traits could be mediated through the effect of mtDNA variation [43, 44]. In addition, the enzyme coded by the *nd5* gene play a vital role in respiratory-chain activities, thus influencing energy supply [45, 46]. Whether the high variation rate of the *nd5* gene identified in the F_1_ groups could be related to phenotypic variation between *MT*×*MA* and *MA*, needs further investigation.

Except for *cox1*, which begins with GTG, all other PCGs initiate with the classical ATG start codon. In most hybrids except *MT*×*MA* and *MS*×*MA*, the stop codons of the 13 PCGs include 7 TAA codons and 6 incomplete stop codons, with 3 TA- (the *atpase6*, *cox3* and *nd4* genes) and 3 T—(the *nd2*, *cox2* and *nd3* genes). In *MT*×*MA* and *MS*×*MA*, the stop codon of the *cytb* gene is a single T (T—), and the stop codons for the other 12 PCGs are the same as other species. It seems that this kind of incomplete termination codon could be fixed by post-transcriptional polyadenylation [47], which is common in vertebrate mitogenomes [37, 48, 49]. There are 2 overlapping reading-frames on the same strand and 1 on the opposite strand. The *atp6* and *atp8* shared 7 bp, the *nd4l* overlapped 7 bp with the *nd4* and the *nd5* shared 4 bp with the *nd6*, which was coded on the opposite strand (Table 6). Gene overlapping is common in mitogenomes of other vertebrate mitogenomes [48, 50–52].

**Table 6:** Characteristics of *M*. *amblycephala* (♀) × *M*. *pellegrini* (♂) mitogenome.

Gene	Position		Size (bp)	Codon		Strand	Intergenic nucleotide (bp)^b^
	From	To		Start	Stop^a^		
D-Loop	1	937	937	-	-	H	0
tRNA-Phe	938	1006	69	-	-	H	0
12S rRNA	1007	1968	962	-	-	H	0
tRNA-Val	1969	2040	72	-	-	H	0
16S rRNA	2041	3733	1693	-	-	H	0
tRNA-Leu	3734	3809	76	-	-	H	0
nd1	3811	4785	975	ATG	TAA	H	1
tRNA-Ile	4790	4861	72	-	-	H	4
tRNA-Gln	4860	4930	71	-	-	L	-2
tRNA-Met	4932	5000	69	-	-	H	1
nd2	5001	6045	1045	ATG	T—	H	0
tRNA-Trp	6046	6116	71	-	-	H	0
tRNA-Ala	6118	6186	69	-	-	L	1
tRNA-Asn	6188	6260	73	-	-	L	1
tRNA-Cys	6293	6360	68	-	-	L	32
tRNA-Tyr	6363	6433	71	-	-	L	2
cox1	6435	7985	1551	GTG	TAA	H	1
tRNA-Ser	8056	7986	71	-	-	L	0
tRNA-Asp	8059	8132	74	-	-	H	2
cox2	8146	8836	691	ATG	T—	H	13
tRNA-Lys	8837	8912	76	-	-	H	0
atpase8	8914	9078	165	ATG	TAA	H	1
atpase6	9072	9754	683	ATG	TA-	H	-7
cox3	9755	10539	785	ATG	TA-	H	0
tRNA-Gly	10540	10611	72	-	-	H	0
nd3	10612	10960	349	ATG	T—	H	0
tRNA-Arg	10961	11030	70	-	-	H	0
nd4l	11031	11327	297	ATG	TAA	H	0
nd4	11321	12702	1382	ATG	TA-	H	-7
tRNA-His	12703	12771	69	-	-	H	0
tRNA-Ser	12772	12840	69	-	-	H	0
tRNA-Leu	12842	12914	73	-	-	H	1
nd5	12915	14750	1836	ATG	TAA	H	0
nd6	14747	15268	522	ATG	TAA	L	-4
tRNA-Glu	15269	15337	69	-	-	L	0
cytb	15342	16478	1137	ATG	TAA	H	4
tRNA-Thr	16483	16554	72	-	-	H	4
tRNA-Pro	16554	16623	70	-	-	L	-1

^a^ T—and T- represent incomplete stop codons;

^b^ Positive numbers refer to the nucleotides separating adjacent genes and negative numbers refer to overlapping nucleotides.

### Ribosomal and transfer RNA genes

All mitogenomes contain two rRNA subunits, 12S rRNA and 16S rRNA, which are separated by tRNA-Val as in the other vertebrates [37]. Twenty variable sites were detected in the rRNA genes of F_1_ groups, with 2 in 12S rRNA and 18 in 16S rRNA. A total of 26 variable sites were observed only in five tRNA genes (4 in tRNA-Phe, 8 in tRNA-Val, 4 in tRNA-Ile, 1 in tRNA-Tyr and 9 in tRNA-His). In the F_2_ generation, 3 variable sites were detected in 16S rRNA and none in 12S rRNA. There were no variable sites found in 22 tRNA genes of all the F_2_ hybrids as well. Twenty-one tRNA genes could be folded into the typical cloverleaf secondary structure [53], with the exception of tRNA-Ser, because the tRNA-Ser (AGY) lacks a DHC arm [40]. In *MA***×***MT*, *MA***×***PP*, *MA***×***MT* and *MA***×***MP*, a variable site in the anticodon was observed in tRNA-Ile. Among tRNA genes, there were 2 overlapping reading-frames on the opposite strand. Two nucleotides overlapped between tRNA-Ile and tRNA-Gln, and one between tRNA-Thr and tRNA-Pro (Table 6) were found in the mitogenomes of all bream and hybrids.

### Non-coding regions

The major non-coding region (D-loop), located between tRNA-Pro and tRNA-Phe genes, was 937 bp in length for most hybrids, except it was 936 bp in *MT*×*MA*. As reported in other fish species [54, 55], 3 conserved domains were identified by multiple homologous sequence alignment, consisting of extended termination associated sequences (ETAS), a central conserved domain (CCD) and conserved sequence blocks (CSB). The terminal associated sequence (TAS) was observed in the first domain with 4 variable sites. It was regarded as the most variable region in the D-loop, which was consistent with other fish species [54]. The central conserved domain was recognized as the most conservative region in the D-loop, containing CSB-F, CSB-E and CSB-D. The third domain, located at the 3’ end of D-loop, was comprised of CSB1, CSB2 and CSB3. The D-loop region is known as an AT-rich region and our data also supported this observation [54]. Based on the structure of the D-loop region reported previously in other fish species [27, 54–57], the conserved motifs in D-loop region of the 17 groups in this study were identified (Table 7).

**Table 7:** The conserved consensus sequence in D-loop region of the 17 bream fishes based on the structure of the D-loop region in other fishes.

Conserved motifs	Consensus sequences^a^
TAS	TACATAT-ATGTATTATCACCAT-ATATTAACCAT
CSB-F	ATGTAGTAAGAGACCACC
CSB-E	AGGG-GTG-GGG
CSB-D	TATTACTTGCAT-TGGCTT-A
CSB-1	ATTATTAAAAGACATA
CSB-2	CAAACCCCCCTACCCCC
CSB-3	TGTCAAACCCC-AAACCAA

^a^-indicates nucleotide variations such as transition, transversion or deletion.

A relatively lower rate of substitution in the D-loop region was found in our study. The mutation rate of the D-loop region (0.30244%) was lower than those of the *nd5* (0.46296%) and *nd2* (0.41467%) genes (Fig 3) in the F_1_ groups. In the F_2_ groups, there were no variable sites found in the D-loop region, the mutation rate of which was lower than those of the *nd1* (0.05128%), *cox1* (0.03224%), *cox3* (0.12739%), *nd4* (0.02412%) and *nd6* genes (0.15964%) (Fig 3).

A non-coding region of 32 bp, the origin of light strand replication (oril) [58], is encoded on L-strand and located between tRNA-Asn and tRNA-Cys. This region has a palindromic sequence, so it can be folded into a stem-loop secondary structure [27]. The conserved motif 5'-GCCGG-3', which was often observed in the tRNA-Cys gene of many vertebrates’ mitogenomes [29, 59, 60], was also detected in our study.

## Conclusions

Our results indicated that, although all the mitogenomes of the hybrids were consistent with maternal inheritance, a certain level of variation was detected in the mitogenomes of F_1_ and F_2_ hybrids, with *MT*×*MA* and *MS*×*MA* groups having relatively more variations. The second generation hybrids showed less mitogenome variation than that of first generation hybrids. The most variable gene in F_1_ groups was the *nd5*, while the *nd6* displayed the highest variability in F_2_ groups. The number of variation sites was found to be related to phylogenetic relationship of the parents. The dN/dS analysis showed that the most PCGs (except the *cox2* and *atp8* genes) were under negative selection. The information reported in this study could be useful for further studies on mitogenome inheritance and variation in fish hybrids.

## References

[1] Saraste M. Oxidative phosphorylation at the *fin de siecle*. Science. 1999; 283(5407): 1488–1493. 10066163 10.1126/science.283.5407.1488

[2] Guo XH, Liu SJ, Liu Q, Liu Y. New progresses on mitochondrial DNA in fish. Acta genetica Sinica. 2004; 31(9): 983–1000. 15493150

[3] Guo XH, Liu SJ, Liu Y. Evidence for maternal inheritance of mitochondrial DNA in polyploid fish of crosses by *ATPase8* and *ATPase6* genes. Acta Zoologica Sinica. 2004; 50(3): 408–413.

[4] Avise JC, Saunders NC. Hybridization and introgression among species of Sunfish (*Lepomis*): analysis by mitochondrial DNA and allozyme markers. Genetics. 1984; 108: 237–255. 6090268 10.1093/genetics/108.1.237 PMC1202397

[5] Gyllensten U, Wharton D, Josefsson A, Wilson AC. Paternal inheritance of mitochondrial DNA in mice. Nature. 1991; 352(6332): 255–257. 1857422 10.1038/352255a0

[6] Kaneda H, Hayashi J, Takahama S, Taya C, Lindahl KF, Yonekawa H. Elimination of paternal mitochondrial DNA in intraspecific crosses during early mouse embryogenesis. Proc Natl Acad Sci USA. 1995; 92(10): 4542–4546. 7753839 10.1073/pnas.92.10.4542 PMC41980

[7] Sutovsky P, Moreno RD, Joã RSO, Dominko T, Simerly Amp C, et al Development: Ubiquitin tag for sperm mitochondria. Nature. 1999; (6760): 371–372. 10.1038/46466 10586873

[8] Xu H, Xiao ZZ, Kong XY, Li J, Dong XL. Analyses of mitochondrial 16S rDNA sequence in *Paralichthys olivaceus* (♀), *P*. *dentatus* (♂) and their hybrids. J Tropical Oceanogr. 2007; 26(5): 60–63.

[9] Li P, Liu L, Tan W, Liu CW. Polymorphism of the mitochondrial DNA D-loop gene sequences of Oreochromis niloticus, O. aureus and their hybrids. J Guangdong Ocean University. 2009; 29(6): 12–15.

[10] Zhou HL, Yang S, Gao C, Zhang L, Zhang HF, Li SS, et al Analysis of genetic variability of mtDNA COI genes between two grouper hybrids and their parents. J Tropical Organisms. 2012; 3(1): 2–10.

[11] Zheng LY. Analysis of maternal inheritance of mitochondrial genes between two crossing grouper hybrids and their parents. J Shanghai Ocean University. 2014; 23(3): 351–358.

[12] Zhu F, Zhang Z, Chen S, Zhang Z, Jin X, Jia C, et al The complete mitochondrial genome of the hybrid of *Pagrus major* (♀) × *Acanthopagrus schlegelii* (♂). Mitochondrial DNA. 2015.

[13] Li X, Wu Z, Zhang G, Zhao C, Zhang H, Qian X, et al The complete mitochondrial genome of the hybrid of *Takifugu fasciatus* (♀) ×* T*. *flavidus* (♂). Mitochondrial DNA Part B Resources. 2016. 10.1080/23802359.2015.1137849 PMC7800279 33490389

[14] Guo XH, Liu SJ, Liu Y. Evidence for recombination of mitochondrial DNA in triploid crucian carp. Genetics. 2006; 172(3): 1745–1749. 16322508 10.1534/genetics.105.049841 PMC1456294

[15] Xu W, Xiong BX. Advances in the research on genus *Megalobrama* in China. J Hydroecology. 2008; 1: 7–11.

[16] Chen J, Li FG, Huang CX, Jiang XY, Zou SM. Morphological variations of genera *Parabramis* and *Megalobrama* teleost populations. J Shanghai Ocean University. 2014; 23(3): 388–394.

[17] Ke H. The artificial reproduction and culture experiment of *Megalobrama amblycephala*. Acta Hydrobiol Sin. 1965; 5: 282–283.

[18] Zhang DL, Du R, Nie ZL, Yang ZH, Luo W, Yi SK, et al The interspecific hybridization in four freshwater bream *Megalobrama* sp. J Dalian Ocean University. 2014; 29(2): 121–125.

[19] Xie G, Ye X, Pang SX, Xu SY, Qi BL. Comparative study on principal genetic characters of the first filial generation (*Megalobrama hoffmanni* ♀ × *Megalobrama amblycephala* ♂) and its Parents. J Hubei Agricultural College. 2002; 22(4): 330–332.

[20] Aquatic Products Bureau of Foushan in Guangdong Province. The hybrids of *M*. *amblycephala* (♀) × *P*. *pekinensis* (♂). Fisheries Science and Technology Information. 1975; 8: 18–19.

[21] Nicholls JA, Double MC, Rowell DM, Magrath RD. The evolution of cooperative and pair breeding in thornbills Acanthiza (Pardalotidae). J Avian Biol. 2000; 31(2): 165–176.

[22] Lowe TM, Eddy SR. tRNAscan-SE: A program for improved detection of transfer RNA genes in genome sequence. Nucleic Acids Res. 1997; 25(5): 955–964. 9023104 10.1093/nar/25.5.955 PMC146525

[23] Yang Z, Nielsen R. Estimating synonymous and nonsynonymous substitution rates under realistic evolution models. Mol Biol Evol. 2000; 17(1): 32–43. 10666704 10.1093/oxfordjournals.molbev.a026236

[24] Yang Z. PAML 4: a program package for phylogenetic analysis by maximum likelihood. Mol Biol Evol. 2007; 24: 1586–1591. 17483113 10.1093/molbev/msm088

[25] Tamura K, Peterson D, Peterson N, Stecher G, Nei M, Kumar S. MEGA5: molecular evolutionary genetics snalysis using maximum likelihood, evolutionary distance, and maximum parsimony methods. Mol Biol Evol. 2011; 28: 2731–2739. 10.1093/molbev/msr121 21546353 PMC3203626

[26] Li SF, Zhu ZW, Zhou SM, Z JL, Cai WQ. Interspecific phylogenesis and intraspecific genetic differences of genus *Megalobrama*: bluntnose black bream (*M*. *amblycephala*), Guangdong black bream (*M*. *hoffmanni*) and black bream (*M*. *terminalis*). Acta zoologica Sinica. 2002; 48(3): 339–345.

[27] Lai RF, Zhang XJ, LI YH, Wu JJ, Yang DH, Wang WM. Comparison of mitochondrial genomes of the genus *Megalobrama* and their phylogenetic analysis. J Fisheries of China. 2014; 38(1): 1–14.

[28] Broughton RE, Milam JE, Roe BA. The complete sequence of zebrafish (*Danio rerio*) mitochondrial genome and evolutionary patterns vertebrate mitochondrial DNA. Genome Res. 2001; 11(11): 1958–1967. 11691861 10.1101/gr.156801 PMC311132

[29] Wang C, Qin C, Lu G, Xu J, Yang Q, Li S. Complete mitochondrial genome of the grass carp (*Ctenopharyngodon idella*, Teleostei): insight into its phylogenic position within Cyprinidae. Gene. 2008; 424(1): 96–101. 18706492 10.1016/j.gene.2008.07.011

[30] Wang XY, Cao L, Liang HW, Li Z, Zou GW. Mitochondrial genome the Short-head catfish (*Pelteobagrus eupogon*). Mitochondrial DNA. 2013; 24(1): 1–2. 10.3109/19401736.2012.710222 22967063

[31] Bolnick DI, Near TJ. Tempo of hybrid inviability in centrarchid fishes (teleostei: centrarchidae). Evolution. 2005; 59(8): 1754–1767. 16329245

[32] Bolnick DI, Turelli M, López-Fernández H, Wainwright PC, Near TJ. Accelerated mitochondrial evolution and "Darwin's Corollary": asymmetric viability of reciprocal F_1_ hybrids in centrarchid fishes. Genetics. 2008; 178(2): 1037–1047. 10.1534/genetics.107.081364 18245356 PMC2248366

[33] Henderson-Arzapalo A, Colura RL, Maciorowski AF. A comparison of black drum, red drum, and their hybrid in saltwater pond culture. J World Aquacult Soc. 1994; 25(2): 289–296.

[34] Kim KK, Bang IC, Kim Y, Nam YK, Kim DS. Early Survival and Chromosomes of Intergeneric Hybrids between Japanese Flounder *Paralichthys olivaceus* and Spotted Halibut *Verasper variegatus*. Fish Sci. 1996; 62(3): 490–491.

[35] Turelli M, Moyle LC. Asymmetric postmating isolation: Darwin's corollary to Haldane's rule. Genetics. 2007; 176(2): 1059–1088. 17435235 10.1534/genetics.106.065979 PMC1894575

[36] Nei M, Kumar S. Molecular evolution and phylogenetics. New York: Oxford University Press 2000.

[37] Wang CH, Wang J, Yang JQ, Lu GQ, Song X, Chen Q, et al Complete mitogenome sequence of black carp (*Mylopharyngodon piceus*) and its use for molecular phylogeny of leuciscine fishes. Mol Biol Rep. 2012; 39: 6337–6342. 10.1007/s11033-012-1455-9 22350152

[38] Kazumasa S, Nobuyuki I, Shinji M, Mitsuto A, Yohey T, Norihiro O, Tachida Hidenori. High prevalence of non-synonymous substitutions in mtdna of cichlid fishes from Lake Victoria. Gene. 2014; 552(2): 239–245. 10.1016/j.gene.2014.09.039 25241383

[39] Qin YH, Chen SY, Lai SJ. Polymorphisms of mitochondrial ATPase 8/6 genes and association with milk production traits in Holstein cows. Anim Biotechnol. 2012; 23(3): 204–212. 10.1080/10495398.2012.686468 22870875

[40] Sutarno CG, Cummins JM, Greeff J, Lymbery AJ. Mitochondrial DNA polymorphisms and fertility in beef cattle. Theriogenology. 2002; 57(6): 1603–1610. 12035972 10.1016/s0093-691x(02)00664-7

[41] Fernando HB, Flávio VM, Ricardo G, Pedro AV, Luiz AFB, Reginaldo AV, et al Mitochondrial DNA single nucleotide polymorphism associated with weight estimated breeding values in Nelore cattle (Bos indicus). Genet Mol Biol. 2007; 30(4): 1058–1063.

[42] Fernández AI, Alves E, Fernández A, Pedro E, Lopez-Garcıa MA, Ovilo C, et al Mitochondrial genome polymorphisms associated with longissimus muscle composition in Iberian pigs. J Anim Sci. 2008; 86(6): 1283–90. 10.2527/jas.2007-0568 18344306

[43] Zhang B, Chen H, Hua LS, Zhang CL, Kang XT, Wang XZ, et al Novel SNPs of the mtDNA nd5 gene and their associations with several growth traits in the Nanyang cattle breed. Biochem Genet. 2008; 46(5–6): 362–368. 10.1007/s10528-008-9152-z 18231850

[44] Suomalainen A, Kaukonen J, Amati P, Timonen R, Haltia M, Weissenbach J, et al An autosomal locus predisposing to deletions of mitochondrial DNA. Nat Genet. 1995; (9): 146–151. 7719341 10.1038/ng0295-146

[45] Anne C. Mitochondrial genetic control of assembly and function of complex I in mammalian cells. J Bioenerg Biomembr. 2001; (33): 251–257. 11695835 10.1023/a:1010791204961

[46] Bai Y, Shakeley RM, Attardi G. Tight Control of respiration by NADH dehydrogenase ND5 subunit gene expression in mouse mitochondria. Mol Cell Biol. 2000; (20): 805–815. 10629037 10.1128/mcb.20.3.805-815.2000 PMC85197

[47] Bibb MJ, Van Etten RA, Wright C T, Walberg MW, Clayton DA. Sequence and gene organization of mouse mitochondrial DNA. Cell. 1981; 26(2): 167–180. 7332926 10.1016/0092-8674(81)90300-7

[48] Liu Y, Cui Z. The complete mitochondrial genome sequence of the cutlassfish *Trichiurus japonicus* (Perciformes: Trichiuridae): Genome characterization and phylogenetic considerations. Mar Genomics. 2009; 2(2):133–142. 10.1016/j.margen.2009.07.003 21798182

[49] Li T, Gao C, Cui Y, Xie Q, Bu W. The complete mitochondrial genome of the stalk-eyed bug *Chauliops fallax* Scott, and the monophyly of Malcidae (Hemiptera: Heteroptera). PLoS One. 2013; 8(2): e55381 10.1371/journal.pone.0055381 23390534 PMC3563593

[50] Chang YS, Huang FL. The complete nucleotide sequence and gene organization of carp (*Cyprinus carpio*) mitochondrial genome. J Mol Evol. 1994; 38(2):138–155. 8169959 10.1007/BF00166161

[51] Chen DX, Chu WY, Liu XL, Nong XX, Li YL, Du SJ, et al Phylogenetic studies of three sinipercid fishes (Perciformes: Sinipercidae) based on complete mitochondrial DNA sequences. Mitochondrial DNA. 2012; 23(2): 70–76. 10.3109/19401736.2011.653799 22409749

[52] Zhou XY, Yu YY, Li YH, Wu JJ, Zhang XJ, Guo XW, Wang WM. Comparative analysis of mitochondrial genomes in distinct nuclear ploidy loach *Misgurnus anguillicaudatus* and its implications for polyploidy evolution. PLoS One. 2014; 9(3): e92033 10.1371/journal.pone.0092033 24643051 PMC3958399

[53] Sprinzl M, Dank N, Nock S, Schön A. Compilation of tRNA sequences and sequences of tRNA genes. Nucleic Acids Res. 1991; 19 (Suppl): 2127–2171. 2041802 10.1093/nar/19.suppl.2127 PMC331350

[54] Guo XH, Liu SJ, Liu Y. Comparative analysis of the mitochondrial DNA control region in cyprinids with different ploidy level. Aquaculture. 2003; 224(1): 25–38.

[55] Zhao JL, Wang WW, Li SF. Structure of the Mitochondrial DNA Control region of the Sinipercine fishes and their phylogenetic relationship. Acta Genetica Sinica. 2006; 33(9): 793–799. 16980125 10.1016/S0379-4172(06)60112-1

[56] Lee WJ, Conroy J, Howell WH, Kocher TD. Structure and evolution of teleost mitochondrial control regions. J Mol Evol. 1995; 41(1): 54–56. 7608989 10.1007/BF00174041

[57] Liu HZ. The structure and evolution of the mtDNA control region in fish: taking example for Acheilognathinae. Prog Nat Sci. 2002; 12(3): 266–270.

[58] Hixson JE, Wong TW, Clayton DA. Both the conserved and divergent 5′-flanking sequences are required for initiation at the human mitochondrial origin of light strand replication. J Biol Chem. 1986; 261(5): 2384–2390. 3944140

[59] Zardoya R., Garrido-Pertierra A, Bautista JM. The complete nucleotide sequence of the mitochondrial DNA genome of the rainbow trout, *Oncorhynchus mykiss*. J Mol Evol. 1995; 41(6): 942–951. 8587139 10.1007/BF00173174

[60] Oh DJ, Kim JY, Lee JA, Yoon WJ, Park SY, Jung YH. Complete mitochondrial genome of the rock bream *Oplegnathus fasciatus* (Perciformes, Oplegnathidae) with phylogenetic considerations. Gene. 2007; 392(1): 174–180. 17258872 10.1016/j.gene.2006.12.007

